# Incidence of Treatment-Limiting Toxicity with Stavudine-Based Antiretroviral Therapy in Cambodia: A Retrospective Cohort Study

**DOI:** 10.1371/journal.pone.0030647

**Published:** 2012-01-27

**Authors:** Vichet Phan, Sopheak Thai, Kimcheng Choun, Lutgarde Lynen, Johan van Griensven

**Affiliations:** 1 Sihanouk Hospital Center of HOPE, Phnom Penh, Cambodia; 2 Institute of Tropical Medicine, Antwerp, Belgium; University of Cape Town, South Africa

## Abstract

**Background:**

Although stavudine (D4T) remains frequently used in low-income countries in Asia, associated long-term toxicity data are scarce. The aim of this study was to determine the long-term incidence of severe D4T-toxicity (requiring drug substitution) and associated risk factors in HIV-infected Cambodians up to six years on antiretroviral treatment (ART).

**Methodology/Principal Findings:**

This is a retrospective analysis of an observational cohort, using data from an ART program with systematic monitoring for D4T-toxicity. Probabilities of time to D4T substitution due to suspected D4T toxicity (treatment-limiting D4T toxicity) were calculated, a risk factor analysis was performed using multivariate Cox regression modelling. Out of 2581 adults initiating a D4T-containing regimen, D4T was replaced in 276 (10.7%) patients for neuropathy, 14 (0.5%) for lactic acidosis and 957 (37.1%) for lipoatrophy. The main early side effect was peripheral neuropathy (7.0% by 1 year). After the first year, lipoatrophy became predominant, with a cumulative incidence of 56.1% and 72.4% by 3 and 6 years respectively. Older age (aHR 1.8; 95%CI: 1.4–2.3) and lower baseline haemoglobin (aHR 1.7; 95%CI: 1.4–2.2) were associated with the occurrence of neuropathy. Being female (aHR 3.8; 95%CI: 1.1–12.5), a higher baseline BMI (aHR 12.6; 95%CI: 3.7–43.1), and TB treatment at ART initiation (aHR 8.6; 95%CI: 2.7–27.5) increased the likelihood of lactic acidosis. Lipoatrophy was positively associated with female gender (aHR 2.3; 95%CI: 2.0–2.6), an older age (aHR 1.3; 95%CI: 1.1–1.4), and a CD4 count <200 cells/µL (aHR 1.3; 95%CI: 1.1–1.5).

**Conclusions:**

Stavudine-based treatment regimens in low-income countries are associated with significant long-term toxicities, predominantly lipoatrophy. Close clinical monitoring for toxicity with timely D4T substitution is recommended. Phasing-out of stavudine should be implemented, as costs allows.

## Introduction

HIV is one of the major health problems in low and middle income countries (LMIC), with over 30 million of people infected. Over the last several years, a successful scaling-up of antiretroviral treatment (ART) has occurred, with currently over five million individuals on treatment. Of these, around 14% live in Asia [1]. The availability of a cheap, generic fixed-dose combination (FDC) has been a key issue in achieving this [2]. In line with WHO recommendations at the start of the ART role-out, almost all national programs have implemented first line treatment consisting of a FDC containing stavudine (D4T), lamivudine and nevirapine [3].

Given increasing reports of D4T-related toxicity, including neuropathy, lactic acidosis and lipoatrophy, WHO 2006 guidelines have recommended to use alternative drugs in stead of D4T [4]. Besides the adoption of an alternative first line regimen for those initiating ART, phasing-out of D4T would additionally require the replacement of D4T for the millions of patients currently using this drug. The recommendation to phase-out D4T was reinforced in the 2010 guidelines [5]. However, recent data demonstrate that the vast majority of individuals in LMIC still take D4T-containing regimens [1]. For a number of reasons, including cost and the operational challenges of the complete phasing-out of D4T, frequent use of D4T-containing regimens will most likely remain the reality on the field for the next years to come. In Cambodia, for instance, the use of D4T-based first line treatment continues up to this day.

Despite the ongoing use of D4T, its recognized potential of toxicity and the maturing of many ART programs [6]–[9], only scarce data on the incidence and timing of its long-term toxicity beyond the first few years on ART are available, particularly from Asian countries. Moreover, studies exploring risk factors of D4T-related toxicity in this region are surprisingly limited. Consequently, it remains unclear which groups might benefit most from any targeted intervention or should be prioritized for phasing-out of D4T. Given the paucity of available data in South-East Asia, including Cambodia, the aim of this study was to determine the long-term incidence of severe D4T-toxicity and associated risk factors in HIV-infected Cambodians. Using data from an ART cohort with systematic monitoring for D4T-toxicity performed from the program-onset in 2003, we provide data on the long-term toxicity associated with D4T by up to six years of treatment.

## Methods

### Study design and study population

We conducted a retrospective cohort study between March 2003 and December 2010 at the Sihanouk-Hospital-Center-of-Hope (SHCH) in Phnom Penh, Cambodia. Since March 2003, this tertiary care hospital provides comprehensive HIV care at no cost, as part of the national ART program. All adult, ART-naive HIV-infected patients having initiating D4T-based ART at SHCH were included.

### Antiretroviral treatment initiation and monitoring

Treatment initiation on ART was according to WHO recommendations: all patients with WHO stage IV, WHO stage III with CD4 cell count <350 cells/µL or with CD4 cell count <200 cells/µL were eligible for ART. First line treatment consisted of a generic FDC containing D4T, lamivudine and nevirapine. Zidovudine or efavirenz was prescribed in case of contraindications to D4T or nevirapine. From 2006 on, D4T was prescribed as 30 mg bid, irrespective of body weight. Prior to that, dosing was done according to body weight with a higher dose (40 mg bid) for individuals with a body weight above 60 kg [4].

Before ART initiation, extensive patient counselling was done, including on the occurrence of toxicities. Patients were seen at monthly visits during the first six months after ART initiation. Subsequently, visits were scheduled less frequently (every 2–3 months) for clinically stable patients. All medical care was provided by physicians, supported by a team of nurses and adherence counsellors. At every clinical encounter, key issues were systematically addressed, including the clinical evolution, evaluation of treatment response and ART-related toxicity. Adherence was evaluated by pill counts at every visit, and using the visual analogue scale (VAS) every six months. Baseline laboratory testing included haematology, liver function tests, hepatitis B/C serology and CD4 cell count determination (FACSCount (Becton Dickinson). After ART initiation, a full blood count and CD4 cell count was done every six months. During the first months of ART, liver function tests were performed more regularly. In case of suspicion of treatment failure, a viral load test was done. Clinical and immunological criteria were used to guide indications for viral load testing. Cotrimoxazole prophylactic treatment was given for all WHO stage II/III/IV patients and all those with a CD4 count <200 cells/µL. All patients with WHO stage 4 disease or a CD4 count <100 cells/µL were started on fluconazole primary prophylaxis. Patients not presenting at their scheduled visit were contacted by phone. Those living in the neighbourhood of the hospital were visited at home. Patients not presenting at the hospital for a period of 6 months without additional information were defined lost to follow-up (LTFU). Additional program details and outcome data of the antiretroviral treatment program in SHCH have been published before [10], [11].

### Outcome measures

All patients on D4T-containing regimens were routinely screened for associated toxicity using a standardized approach. At every clinic visit, patients were assessed for symptoms suggestive of symptomatic hyperlactatemia or lactic acidosis. Lactate level was measured for suspected cases only. Since no pH determination could be done, cases of symptomatic hyperlactatemia (SH) and lactic acidosis (LA) could not be differentiated and were grouped together as SH/LA. A case of SH/LA was defined as a patient on ART, presenting with compatible symptoms with other mimicking conditions ruled out, and a lactate level ≥2.5 mmol/L (capillary blood, Accutrend Lactate). D4T was systematically replaced for these patients. Up to 2005, no lactic acid levels could be measured and diagnosis was clinical. Neuropathy was assessed clinically by the HIV physician, D4T was substituted in case of WHO grade III/IV severity. Patients were assessed for lipoatrophy (loss of subcutaneous fat in the face, arms, legs, cheeks, buttocks) at every encounter by self-report and clinical assessment. Decisions to substitute D4T were guided by the clinical severity of lipoatrophy combined with the patient's preference and perception of the body changes. Consequently, stavudine was essentially replaced for reasons of lipoatrophy if 1) clinically severe or 2) patients perceived this as severe and disturbing, even though clinically it was defined as less pronounced.

### Data collection and statistical analysis

Clinical and laboratory data were prospectively collected on a daily basis, using standardized data collection tools and stored in a database. Quality control of the stored data was done at regular intervals.

The primary outcome was time to D4T substitution due to suspected D4T toxicity. We will refer to this in the text as treatment-limiting (or severe) D4T toxicity. The cumulative incidence of D4T substitution due to suspected toxicity was calculated using the Kaplan Meier methodology. Patients were censored at the date of D4T substitution, at the last visit for patients that died, were transferred out or were lost to follow-up, and at December 31, 2010 for the remainder. Patients switched to non-D4T containing second line regimens due to virological failure were censored at the date of switching and defined as having experienced no D4T-related toxicity event. A risk factor analysis was performed using multivariate Cox regression. Collinearity between variables was assessed. We used a back-ward selection method, retaining those variables with *P*-values<0.05 in the final model. Data analysis was done using STATA version 11. The level of significance was set at *P*<0.05.

### Ethical issues

Since the launch of the HIV care program, clinical data have been routinely collected for purposes of program monitoring and evaluation, and research activities. Patients were requested to give written informed consent to store and use the data. No linkage of these data with other sources was done. The data collection and informed consent procedure were approved by the Institutional Review Board ITM (Institute of Tropical Medicine, Antwerp) and the Institutional Review Board SHCH (Sihanouk Hospital Center of HOPE). No patient identifiers were included in the dataset used for this analysis.

## Results

### Characteristics of the study population

By December 2010, 2581 adult patients on D4T-containing regimen were included in analysis, with a median follow-up time of 1.3 years. Of these, 1341 (52%) were female, the median age at the start of treatment was 35 years. Eighty percent were in WHO stage III/IV at treatment initiation. The median baseline CD4 count was 87 cells/µL. The most frequent ART regimen was D4T/lamivudine/nevirapine, with 72% of patients initiating this regimen. In total, 686 (27%) of the patients were on TB treatment at ART initiation (Table 1).

**Table 1:** Baseline characteristics of adult patients initiating stavudine-containing antiretroviral treatment (N = 2581).

Age (years) - median (IQR)	35 (30–40)
Sex - n (%)	
Male	1240 (48)
Female	1341 (52)
Baseline WHO clinical stage - n (%)	
Stage I	127 (4.9)
Stage II	404 (15.7)
Stage III	1081 (41.9)
Stage IV	969 (37.5)
Baseline body weight (kg) – median (IQR)	49 (43–55)
Baseline body mass index (kg/m^2^) - median (IQR)	19 (17–21)
Baseline CD4 count (cells/µL) - median (IQR)	87 (25–206)
Baseline haemoglobin (g/dL) - median (IQR)	11.3 (9.9–12.6)
Treatment regimen - n (%)	
Stavudine/lamivudine/nevirapine^a^	1869 (72.5
Stavudine/lamivudine/efavirenz^a^	719 (27.5)
On tuberculosis treatment at ART initiation - n (%)	686 (26.6
Follow-up time with exposure to D4T (years) - median (IQR)	1.3 (0.8–2.3)

IQR: interquartile range, WHO: world health organization, ART: antiretroviral therapy.

a From 2006, stavudine was prescribed as 30 mg bid, irrespective of body weight. Prior to that, 40 mg bid was given for individuals with a body weight >60 kg.

### Incidence and timing of D4T-related toxicity

**Figure 1:**
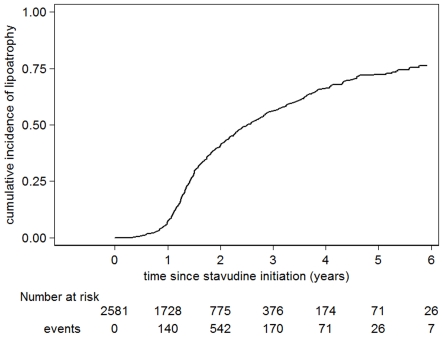
Cumulative incidence of lipoatrophy. Kaplan-Meier curve showing the proportion of patients substituting stavudine due to lipoatrophy.

Out of 2581 adult patients initiating a D4T-containing regimen, D4T was replaced in 276 (10.7%) patients for suspected D4T-related neuropathy, 14 (0.5%) for SH/LA and 957 (37.1%) for lipodystrophy. All reported cases of SH/LH were confirmed by lactic acid determination. The main early side effect was peripheral neuropathy (7.0% by 1 year), with a cumulative incidence of 16.6% and 19.0% by 3 and 6 years respectively. SH/LA was mainly seen after the first six months but remained rare overall, with a cumulative incidence of 1% by 6 years. After the first year, lipoatrophy became the predominant side effect, with a cumulative incidence of 56.1% and 72.4% by 3 and 6 years respectively (Table 2; Figure 1).

**Table 2:** Incidence of severe toxicity related to stavudine (N = 2581).

Stavudine-related toxicity	Events (%)	Rate/1000 py	Cumulative incidence (%) – Kaplan-Meier estimates^a^			
			6 m	12 m	36 m	60 m
Neuropathy	276 (10.7)	63.8	2.1	7.0	16.6	19.0
Lactic acidosis	14 (0.5)	3.2	0.1	0.6	0.7	1.0
Lipoatrophy	957 (37.1)	221.1	0.8	7.0	56.1	72.4

a Kaplan-Meier estimates: time to first severe toxicity (ie requiring treatment change) at specified months on ART; py:: patient years.

### Risk factors for D4T-related toxicity

In univariate analysis, older age (>40 years), low baseline body mass index (BMI) (<18.5 kg/m^2^), and low baseline haemoglobin (<10 g/dL) were associated with increased risk of D4T-associated neuropathy (Table 3). Higher baseline BMI (>25 kg/m^2^), use of efavirenz and being on TB treatment at ART initiation were associated with increased risk of SH/LA. Lipoatrophy was positively associated with older age and female sex. An increased risk was observed with low baseline CD4 cell count (<200 cells/µL) and low baseline haemoglobin. In multivariate analysis, neuropathy remained associated with older age and low baseline haemoglobin. Being female, overweight (BMI>25 kg/m^2^), and on TB treatment at ART initiation increased the likelihood of SH/LA. Lipoatrophy was positively associated with female gender and older age. A reduced risk was seen for patients initiating ART with a baseline CD4 count >200 cells/µL (Table 3).

**Table 3:** Risk factors for severe toxicity related to stavudine.

Risk factors	Neuropathy		Lactic acidosis		Lipoatrophy	
	HR (95% CI)	aHR (95% CI)	HR (95% CI)	aHR (95% CI)	HR (95% CI)	aHR (95% CI)
Sex						
Male	1		1	1	1	1
Female	1.1 (0.9–1.4)		2.5 (0.8–8.1)	3.8 (1.1–12.5)	2.2 (1.9–2.5)	2.3 (2.0–2.6)
Age						
≤40 years	1	1	1		1	1
>40 years	1.9 (1.5–2.4)	1.8 (1.4–2.3)	2.8 (1.0–8.1)		1.1 (1.0–1.3)	1.3 (1.1–1.4)
Baseline WHO clinical stage						
I/II	1		1		1	
III/IV	1.4 (1.0–2.0)		1.5 (0.3–6.7)		1.0 (0.9–1.2)	
Baseline BMI						
≤25 kg/m^2^	-		1	1	1	
>25 kg/m^2^	-		6.4 (2.0–20.5)	12.6 (3.7–43.1)	0.9 (0.7–1.3)	
≥18.5 kg/m^2^	1		-	-	-	-
<18.5 kg/m^2^	1.3 (1.0–1.7)		-	-	-	-
Baseline CD4 count						
≥200 cells/µL	1		1		1	1
<200 cells/µL	1.1 (0.8–1.5)		0.8 (0.3–2.6)		1.2 (1.0–1.4)	1.3 (1.1–1.5)
Baseline haemoglobin						
≥10 g/dL	1	1	1		1	
<10 g/dL	1.7 (1.4–2.2)	1.7 (1.4–2.2)	1.3 (0.4–4.3)		1.2 (1.0–1.3)	
NNRTI at start						
EFV	1		1		1	
NVP	0.9 (0.7–1.1)		0.3 (0.1–0.7)		0.9 (0.8–1.1)	
On TB treatment at ART start						
No	1		1	1	1	
Yes	1.1 (0.9–1.5)		5.4 (1.8–16.0)	8.6 (2.7–27.5)	1.0 (0.9–1.2)	

aHR: adjusted hazard ratio, CI: confidence interval, WHO: world health organization, BMI: body mass index, NNRTI: non-nucleoside reverse transcriptase inhibitor; EFV: efavirenz; NVP: nevirapine; TB: tuberculosis; ART: antiretroviral treatment.

## Discussion

This paper is the first to provide estimates of long-term toxicity related to D4T up to six years after treatment initiation from LMICs. By six years on D4T, lipoatrophy was the predominant side-effect, requiring treatment substitution in seven out of ten patients. For each of the different D4T-toxicities, distinct risk factors were identified, indicating which patients might require more close toxicity-monitoring, should avoid D4T altogether or should be prioritized for phasing-out of D4T.

Our findings of older age and female sex as risk factors for lipoatrophy are consistent with other reports from LMIC [12], [13]. Regarding baseline CD4 cell count, conflicting data have been reported [12], [13]. Our data confirm the reported association of older age and neuropathy [12], [14], [15]. Whereas most previous studies have reported advanced HIV stage as a risk factor for neuropathy, we observed an increased risk associated with low baseline haemoglobin. Possibly, this is merely a reflection of advanced disease at ART initiation [10], [13]. Alternatively, recent studies have observed the strong and independent prognostic information contained in both baseline and time-updated haemoglobin levels, even after adjustment for CD4 cell count values [10], [11], [16]. In contrast with a South African study [17], we did not find an association of neuropathy with TB treatment. Possible reasons for this could include differences in analytical approach, management of toxicity or timing of ART initiation for patients on TB treatment.

With regards to SH/LA, the association with the use of tuberculosis treatment is somehow surprising and has not been reported yet. In one case-control study, efavirenz was identified as a risk factor for lactic acidosis [18]. Whether this could be the mechanism behind our observed association with tuberculosis treatment, albeit not identified in multivariate analysis, remains to be determined. Alternatively, rapid weight gain after ART initiation, identified as a risk factor for lactic acidosis in a South African study, might be implicated for patients on tuberculosis treatment [12]. Finally, it needs to be pointed out that TB treatment and the use of EFV are closely related variables. Although no clear problem of collinearity was detected during analysis, the problem cannot be entirely ruled out.

Cost has been a major reason for the ongoing use of D4T-containing ART in LMIC. Despite recent cost reductions, tenofovir-based regimens are still more than twice as expensive in terms of drug costs. In Cambodia, the low cost of D4T is a key argument for maintaining this drug within first line treatment regimens for the next years to come. Our data suggest that the cost-saving effect with D4T-use is limited in time, given the high long-term rates of D4T-replacement. Moreover, its ongoing use continues to expose patients to drug toxicity, with all its negative implications. Whereas our data reinforce the need to phase-out D4T to better tolerated regimens in LMIC, it is clear that this is a major operational undertaking that should be implemented in a phased and controlled manner. In this regard, our experience could be of interest for national programs willing to implement a gradual phasing-out of D4T. By combining patient education, close monitoring for D4T-toxicities, integrating the patient's perception and applying a low threshold for D4T-replacement, a gradual phasing-out of D4T can be expected. Additionally, the occurrence of toxicity could be significantly reduced by prioritizing those at highest risk of D4T toxicity, based on the risk factors identified. Importantly, patient support relative to a uniform and quick D4T replacement strategy might be enhanced with this more targeted approach. A recent study from Cambodia on systematic substitution of zidovudine for D4T highlighted that a fraction of patients preferred to remain on D4T-based HAART [19]. Imposing a treatment change without patient approval might negatively affect on the adherence to the new regimen.

Previous studies have reported on D4T-toxicity up to three years after ART initiation [12], [15]. Although incidence of neuropathy in our study was lower, our three-year estimates on lipoatrophy are around twice those recently reported in an African cohort [15]. This could in part relate to the strict criteria for D4T-substitution in this program, relying on a scoring-system of clinical severity of changes in different body sites. In contrast, we also considered the patient's perception and preference. Indeed, recent evidence is consistently pointing out that the subjective experience of ART-related toxicities might be as an important parameter to monitor in ART programs besides the ‘objective’ changes [20]–[23]. Self-reported physical and psychological symptoms were identified as strong and independent risk factors for subsequent treatment failure in a recent study [24]. From an operational perspective, the patient's perception is probably an integral part in quantifying the (‘subjective’) severity of toxicities. Integrating this in therapeutic decisions could contribute towards greater adherence to proposed interventions and towards improvement in the quality of life. This might be especially true for toxicities like body changes, where clear inter-individual differences in perception could exist. The negative impact of perception of body changes on quality of life and adherence has been reported in a number of studies [25]–[28].

A number of limitations have to be mentioned. This is a retrospective analysis, using data from a treatment program setting. Data on D4T dosing were not recorded in the database. However, given the overall low body weight in this population, few patients initiated high dose D4T (40 mg bid) before revision of the guidelines in 2006. D4T drug changes were driven by suspicion of D4T toxicity by the clinician, hence not necessarily with formal proof of D4T as the culprit drug. Reported data merely demonstrate associations, and not causation. Moreover, this program was probably better resourced with more intensive monitoring compared to other field settings, and this could possibly have resulted in over diagnosis of toxicities. With increasing experience of the program, diagnosis may have been influenced by negative perceptions about D4T by physicians and patients. However, patients were systematically evaluated at each clinical visit, with standard clinical assessment, patient management and reporting. We also note that data were collected prospectively using standardized data collection tools, with continuous monitoring of data quality and clinical management practices. Still, it remains that the unavailability of technical investigations to more rigorously diagnose the different toxicities could have led to misclassification. Moreover, at the start of the program, no lactic acid determination could be done. For lipoatrophy, diagnostic tools such as DEXA-scanning might have allowed to define the exact incidence of lipoatrophy over time. However, our main research question was not to define what happens ‘biologically’, but rather ‘operationally’ ie to what extent and for what reasons D4T is replaced over time within a program with close toxicity monitoring.

D4T-based treatment regimens in low-income countries are associated with significant long-term toxicities. With seven out of ten patients developing lipoatrophy by six years of treatment, lipoatrophy represents the major long-term side-effect. Until D4T has been phased-out completely, close monitoring for toxicity combined with the integration of the patient's perspective and a low threshold for D4T-replacement is recommended.
